# American Electro-Therapeutic Association

**Published:** 1899-11

**Authors:** 


### American Electro-Therapeutic Association.

The ninth annual meeting of the American Electro-Therapeutic Association, was held in Washington, D. C., at Willard's Hotel, on September 19, 20 and 21, 1899. The convention was very successful, in point of attendance and interest, and a number of papers were read and discussed of scientific value and importance to the medical profession. The program included thirty-six papers and the reports of seven standing committees on scientific questions relating to the medical application of the electrical current, with the best electrical apparatus extant. The proceedings of the convention will be found in their annual transactions, to be published at an early date. The officers elected for the tenth year, are:

President, Walter H. White, M. D., Boston, Mass.; First Vice-President, D. Percy Hickling, M. D., Washington, D. C.; Second Vice-President, Charles O. Files, M. D., Portland, Me.; Treasurer, Richard J. Nunn, M. D., Savannah, Ga.; Secretary, George E. Bill, M. D., Harrisburg, Pa.

The next annual meeting will be held in New York City on September 25, 26 and 27, 1900.

